# Relatively lower body mass index is associated with an excess of severe truncal asymmetry in healthy adolescents: Do white adipose tissue, leptin, hypothalamus and sympathetic nervous system influence truncal growth asymmetry?

**DOI:** 10.1186/1748-7161-4-13

**Published:** 2009-06-30

**Authors:** Theodoros B Grivas, R Geoffrey Burwell, Constantinos Mihas, Elias S Vasiliadis, Georgios Triantafyllopoulos, Angelos Kaspiris

**Affiliations:** 1 Department of Trauma and Orthopaedics, Scoliosis Clinic, "Thriasio" General Hospital – NHS, G. Gennimata Av. 19600, Magoula, Attica, Greece; 2 Department of Trauma and Orthopaedics, "Tzanio" General Hospital – NHS, Tzani & Afendouli str, 18536, Piraeus, Greece; 3 Centre for Spinal Studies & Surgery, Nottingham University Hospitals Trust, Queen's Medical Centre Campus, Nottingham NG7 2UH, UK

## Abstract

**Background:**

In healthy adolescents normal back shape asymmetry, here termed truncal asymmetry (TA), is evaluated by higher and lower subsets of BMI. The study was initiated after research on girls with adolescent idiopathic scoliosis (AIS) showed that higher and lower BMI subsets discriminated patterns of skeletal maturation and asymmetry unexplained by existing theories of pathogenesis leading to a new interpretation which has therapeutic implications *(double neuro-osseous theory)*.

**Methods:**

5953 adolescents age 11–17 years (boys 2939, girls 3014) were examined in a school screening program in two standard positions, standing forward bending (FB) and sitting FB. The sitting FB position is thought to reveal intrinsic TA free from back humps induced by any leg-length inequality. TA was measured in both positions using a Pruijs scoliometer as angle of trunk inclinations (ATIs) across the back at each of three spinal regions, thoracic, thoracolumbar and lumbar. Abnormality of ATIs was defined as being outside 2 standard deviations for each age group, gender, position and spinal region, and termed *severe *TA.

**Results:**

In the sitting FB position after correcting for age,*relatively lower BMIs *are statistically associated with a greater number of severe TAs than with relatively higher BMIs in both girls (thoracolumbar region) and boys (thoracolumbar and lumbar regions).

The relative frequency of severe TAs is significantly higher in girls than boys for each of the right thoracic (56.76%) and thoracolumbar (58.82%) regions (p = 0.006, 0.006, respectively). After correcting for age, smaller BMIs are associated with more *severe TAs *in boys and girls.

**Discussion:**

BMI is a surrogate measure for body fat and circulating leptin levels. The finding that girls with relatively lower BMI have significantly later menarche, and a significant excess of TAs, suggests a relation to energy homeostasis through the hypothalamus. The hypothesis we suggest for the pathogenesis of severe TA in girls and boys has the same mechanism as that proposed recently for AIS girls, namely: severe TAs are initiated by a *genetically-determined selectively *increased hypothalamic sensitivity (up-regulation, i.e. increased sensitivity) to leptin with asymmetry as an adverse response to stress (hormesis), mediated bilaterally mainly to the growing trunk via the sympathetic nervous system *(leptin-hypothalamic-sympathetic nervous system (LHS) concept)*. The putative autonomic dysfunction is thought to be increased by any lower circulating leptin levels associated with relatively lower BMIs. Sympathetic nervous system activation with asymmetry leads to asymmetries in ribs and/or vertebrae producing severe TA when beyond the capacity of postural mechanisms of the somatic nervous system to control the shape distortion of the trunk. A test of this hypothesis testing skin sympathetic responses, as in the Rett syndrome, is suggested.

## Introduction

The value of school screening for scoliosis has largely been an appreciation of the high prevalence of small degrees of surface deformity and curvature and the opportunity to study the natural history of these curvatures [1,2]. Not adequately recognised is the potential, through epidemiological studies of screened subjects, to understand better some factors involved in the pathogenesis of adolescent idiopathic scoliosis (AIS) [3-13]. In this connection, body mass index (BMI) has been evaluated in relation to both AIS [14-23] and the trunk asymmetry (TA) of normal subjects [7,16,23-25]. But no attempts have been made to evaluate how BMI may relate to the pathogenesis of the trunk shape distortion of TA as it has recently for girls with AIS [23].

Employing a new approach of comparing skeletal data between subsets of higher and lower body mass index (BMI) in adolescent girls – preoperative with AIS, screened for AIS and normals, BMI was found to be related to skeletal maturation of the trunk, and to some skeletal asymmetries of AIS [23]. The findings were not explained by any of the theories of pathogenesis for AIS surveyed in a recent review [26]. A novel pathogenetic interpretation was formulated as a *double neuro-osseous theory*, involving disharmony between the autonomic and somatic nervous systems expressed in the spine and trunk with therapeutic implications [27-30].

In normal adolescents, we test whether such BMI subsets are related to severe TAs. To prepare our TA data for analysis, the known compounding factor of any leg-length inequality on back shape was removed. This was done by basing the interpretation on TA readings obtained from subjects in the sitting FB position, because when a healthy child is examined in the standing FB position, shortness of one leg causes a contralateral hump on the back [31-40]. Previous research suggested that the sitting FB position expresses intrinsic TAs free from extrinsically-induced effects of any leg-length inequality [34,41-43].

Normal TAs and AIS form a continuum of TAs with thresholds needed to prescribe abnormality [1,2,37,42]. In a 3-year longitudinal study of TA in healthy prepubertal school children, scoliosis curves of 10 degrees or more occurred in 9.5% of girls and 5.1% of boys [24]. The biological mechanisms that cause TA are ill-understood but are likely to have some common ground with the pathogenesis of AIS, where genetic factors and the fully upright human spine may play decisive roles [44]. In premenarcheal girls, left truncal humps [41,42,45] and left scoliosis curves [6] are more prevalent than are right humps, or curves. In postmenarcheal girls, right humps become prevalent.

Skeletal imaging studies have examined TAs in relation to:

• rib-vertebra angle asymmetry [46,47];

• convex/concave rib-hump index ('double rib contour sign') [9,48];

• vertebral sagittal profile [26,49,50]; and

• ultrasound axial vertebral and rib rotations [51] viewed in relation to radiographic axial vertebral rotation [26,51-53] and spine-rib rotation difference [54].

Changes in TA with age in normal subjects leading to AIS have been interpreted as resulting from growth with the rib cage deformity preceding the spinal deformity [48].

The aims of this paper in normal girls and boys are firstly, to examine the relationship between (1) severe trunk asymmetry, (2) menarche and (3) two BMI subsets – relatively higher and lower than median values for age, sex, and spinal region and secondly, to interpret the findings from the standpoint of TA pathogenesis. A preliminary report has been presented [55].

## Methods

### The healthy children

Throughout the school period (September to June) from 1996 to 2007, 5,953 children (2939 boys with a mean age 13.39 ± 1.48 and 3014 girls with a mean age 13.39 ± 1.48 years), were examined during the school screening program of the hospital (Table 1).

**Table 1:** Age distribution of the examined children by gender. Gender frequency does not change among age groups, (chi-square for trend p = 0.880)*.

		Gender				
		
		Boys		Girls		Count
			
		Count	Row %	Count	Row %	
Decimal Age	11	**595**	*49.5%*	**608**	*50.5%*	**1203**
categories						
	12	**678**	*49.7%*	**686**	*50.3%*	**1364**
	
	13	**677**	*47.6%*	**745**	*52.4%*	**1422**
	
	14	**586**	*50.6%*	**571**	*49.4%*	**1157**
	
	15	**239**	*52.8%*	**214**	*47.2%*	**453**
	
	16	**112**	*46.7%*	**128**	*53.3%*	**240**
	
	17	**52**	*45.6%*	**62**	*54.4%*	**114**

Total		**2939**	*49.4%*	**3014**	*50.6%*	**5953**

* There are missing values of some variables so the same numbers (absolute frequencies) are not the same in all Tables.

### Examination and measurements (Figures 1 &2)

**Figure 1:**
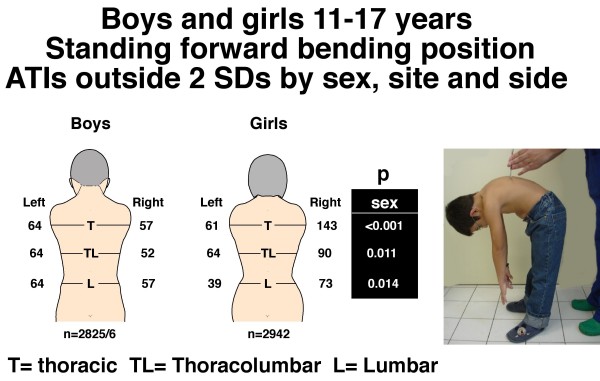
**Boys and girls 11–17 years**. Standing forward bending position. Angle of trunk inclinations (ATIs), number outside ± 2 standard deviations by sex, site and side. Note the difference of the number of severe TAs between boys and girls with P values by region.

**Figure 2:**
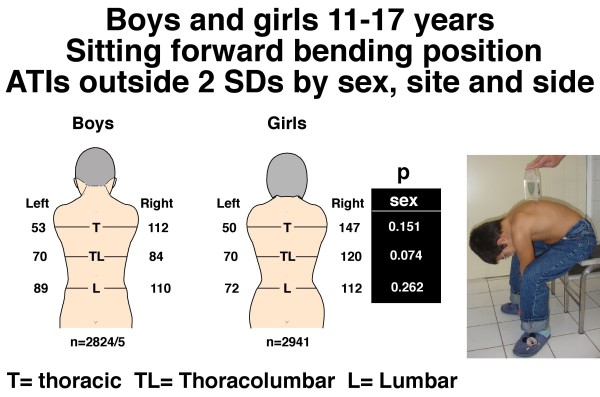
**Boys and girls 11–17 years**. Sitting forward bending position. Angle of trunk inclinations (ATIs), number outside ± 2 standard deviations by sex, site and side. Note there is no difference of the number of severe TAs between boys and girls with P values by region. But for the right thoracic region, the relative frequency of severe TAs is significantly higher in girls than boys (respectively 5.08% and 4.04%, p = 0.006) and right thoracolumbar region (respectively 4.18% and 4.04%, p = 0.023).

TA was measured in two standard positions, standing FB and sitting FB. TA was assessed using a Pruijs Scoliometer (Orthomet-Surgeyplant B.V. Waalwijl, Netherlands) [56]) by measuring angles of trunk inclination (ATIs) in degrees at each of thoracic (T4-T8), thoracolumbar (T12-L1) and lumbar (L2-5) regions of the back. The child was asked to bend forwards, look down, keeping the feet 15 cm apart, knees braced back, shoulders loose, and hands positioned in front of the knees or shins with elbows straight and palms apposed (Figure 1). Any detected leg-length inequality was not corrected. The side of the hump determined the laterality of the trunk inclination, humps on the right (positive) and on the left (negative) in each of the three spinal regions. In the sitting FB position (Figure 2), the student was seated on a chair about 40 cm high, and asked to bend forwards and place the head between the knees with shoulders loose, elbows straight and hands positioned between the knees. The scoliometer measurements were obtained successively at the three spinal regions as in the standing FB position. The reproducibility of the scoliometer measurements has been reported [8].

### Stature

The standing height was measured using a stature meter in cm.

### Weight

Body weight was assessed using a weight scale with 0.5 kg increments.

### Body mass index (BMI)

BMI was calculated as body weight in kg/stature in meters^2^.

### Stature SD scores

Stature SD scores (SDSs), or Z scores, were calculated as the difference between the observed (y) and the mean value (Y) for age divided by the standard deviation (SD) of the mean for each age year and sex. SDS (SDS = Z score) = (y-Y)/SD

### Grouping of ATIs

#### First method – by absolute numbers

At each of the three spinal regions on the child's back in each of the standing and sitting FB positions, group 0 consisted of children who were symmetric; group 1 children with ATIs 1–6°; and group 2 the ATI was 7° or more [16].

#### Second method – outside 2 standard deviations

Children were categorized in two groups: Group a) mean ATI ± 2 standard deviations (SDs), and group b) ATIs higher or lower than the aforementioned value range termed here outside or beyond 2 SDs (> or <), or severe TA. The last group was also transformed by laterality into two subgroups each beyond 2 SDs, group *brt *and group *bl*, with TA right and left respectively.

### Grouping of BMI – above and below median values

A BMI *median value *was constructed for each age group, gender, position and spinal region, and each child assigned a value of *relatively "lower" or relatively "higher" BMI subset *in relation to the median values.

### Menarcheal age and BMI

The mean age at which girls had their menarche was calculated for both higher and lower BMI subgroups as were the numbers of pre- and post-menarcheal girls. Other assessments of sexual maturity are not available for the girls. Sexual maturity findings were not obtained from the boys.

### Statistical analysis & data processing

The scoliometer ATI readings were analysed by spinal region, sex, side, age and BMI subset.

In order to test for any independence between categorical variables, tables were constructed and the Pearson Chi-squared test was used.

Continuous variables are shown as means and standard deviations (SDs).

Stature SDSs were plotted *versus *ATIs for each gender and spinal region (thoracic, thoracolumbar, lumbar) in both standing and sitting FB positions in order to investigate any association between TA and age with the Pearson's correlation coefficients being calculated.

The corresponding univariate linear regression models of ATIs on stature SDs were also constructed for the same purpose.

Statistical tests were considered significant if p values are less than 0.05

The data were analysed using STATA™ (Version 9.0, Stata Corporation, College station, TX 77845, 800-782-8272).

## Results

### Body mass index in the normal boys and girls

**Figure 3:**
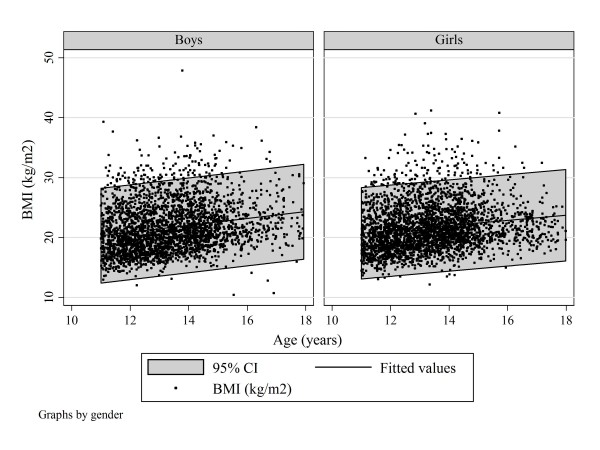
**All subjects**. Scatter plot of BMI by age and gender to show best-fit linear regression and 95% confidence intervals.

Table 2 shows the BMI distribution by age, gender, trunk symmetry and severe trunk asymmetry. Figure 3 shows the BMI scatter by age in the entire samples of boys and girls.

**Table 2:** Subjects with symmetry of back shape (normal) and severe trunk asymmetry.

	**Boys**			
	**Normal**		**Trunk asymmetry**	
**Age**	**Mean**	**SD**	**Mean**	**SD**
11	20.66	4.09	19.18	3.29
12	21.39	4.13	20.67	4.03
13	22.00	4.20	21.32	3.40
14	22.70	4.04	21.96	3.90
15	22.73	3.73	22.34	3.94
16	22.51	4.05	25.74	5.02
17	23.72	4.01	22.52	2.72
	
	**Girls**			
	
11	20.57	3.84	20.33	3.24
12	21.55	4.01	20.37	3.77
13	22.44	4.19	21.20	3.24
14	22.72	3.86	21.66	3.40
15	22.50	3.69	22.44	4.39
16	22.40	3.21	22.04	3.44
17	22.15	2.85	20.90	2.41

BMI distribution by age, gender and back shape.

### Body mass index in students with severe trunk asymmetry

**Figure 4:**
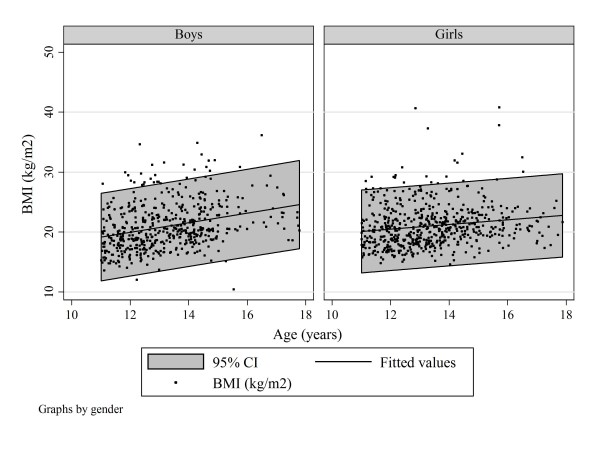
**Subjects with severe trunk asymmetry**. Scatter plot of BMI by age and gender to show best-fit linear regression and 95% confidence intervals.

**Figure 5:**
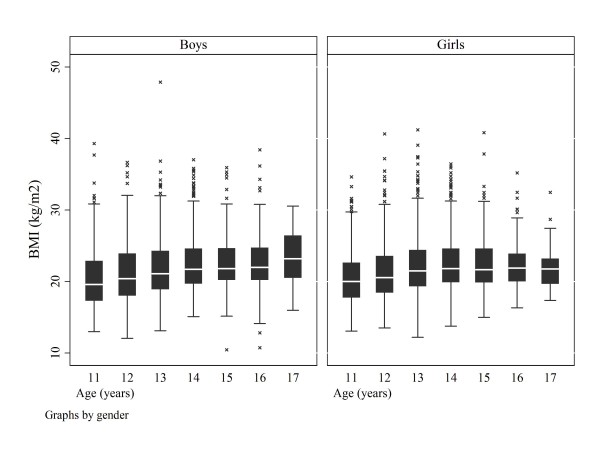
**Subjects with severe trunk asymmetry**. Box plots of BMI by age and gender

Figure 4 shows the BMI scatter by age and Figure 5 the boxplots of BMIs for the boys and girls with severe trunk asymmetry by age.

### Percentage of TAs outside 2 standard deviations – standing and sitting FB positions

(Table 3). In the standing FB position, using 2 standard deviations or more to define severe (abnormal) TA, there were: boys, thoracic 4.3%, thoracolumbar 4.1%, lumbar 4.3%; and girls, thoracic 5.9%, thoracolumbar 5.5%, lumbar 7.1%. In the sitting FB position, using 2 standard deviations or more to define severe (abnormal) TA, there were: boys, thoracic 5.8%, thoracolumbar 5.4%, lumbar 7.0%; and girls, thoracic 6.7%, thoracolumbar 6.5%, lumbar 6.3%.

**Table 3:** Subjects with symmetry of back shape (Mean +/- 2 SD) and severe trunk asymmetry (Beyond +/- 2 SD).

		**Boys**				
		**Standing**		**Sitting**		
		**Count**	**Col %**	**Count**	**Col %**	***p***
**ATI T**	**Mean +/- 2 SD**	2696	*95.70*	2651	*94.14*	0.008
	**Beyond +/- 2 SD**	121	*4.30*	165	*5.86*	

**ATI T-L**	**Mean +/- 2 SD**	2701	*95.88*	2663	*94.53*	0.018
	**Beyond +/- 2 SD**	116	*4.12*	154	*5.47*	

**ATI L**	**Mean +/- 2 SD**	2696	*95.70*	2617	*92.93*	<0.001
	**Beyond +/- 2 SD**	121	*4.30*	199	*7.07*	

		**Girls**				

**ATI T**	**Mean +/- 2 SD**	2726	*93.04*	2732	*93.27*	0.720
	**Beyond +/- 2 SD**	204	*6.96*	197	*6.73*	

**ATI T-L**	**Mean +/- 2 SD**	2776	*94.74*	2739	*93.51*	0.045
	**Beyond +/- 2 SD**	154	*5.26*	190	*6.49*	

**ATI L**	**Mean +/- 2 SD**	2818	*96.18*	2746	*93.75*	<0.001
	**Beyond +/- 2 SD**	112	*3.82*	183	*6.25*	

Distribution of trunk asymmetry (TA) as angles of trunk inclination (ATIs) by forward bending (FB) position, spinal region and gender.

### Severe TA – comparison by sex in standing and sitting FB positions

Figures 1 and 2 show the number of boys and girls with ATIs outside 2 SDs in the standing and sitting FB positions for each of the three regions, thoracic, thoracolumbar and lumbar. In the standing FB position the girls are more asymmetric (6.93% vs. 4.28%, 5.24% vs. 4.10%, for thoracic and thoracolumbar regions, respectively), whereas *overall *in the sitting FB position no statistically significant difference for severe TA was observed between genders.

### Severe TA – comparisons by standing and sitting FB positions in boys and girls

With the exception of the thoracic region in girls, the distribution of TA differed significantly between standing and sitting position in the other spinal regions of the two genders (Table 3). More specifically, TA beyond 2 SDs was always more prevalent in the sitting than in the standing FB position in all spinal regions except the thoracic region of girls.

### Severe TA – comparisons by side for sex

(Table 4) In the standing FB position, the number of boys and girls with ATIs outside 2 SDs collectively for the three regions, thoracic, thoracolumbar and lumbar, is not significantly different between left and right (boys p = 0.921, girls (p = 0.072). In the sitting FB position, there is an excess of right relative to left ATIs outside 2 SDs in both boys (p = 0.020) and girls (p = 0.009); and the relative frequency of severe TAs is significantly higher in girls than boys for the right thoracic region (respectively 5.08% and 4.04%, p = 0.006) and right thoracolumbar region (respectively 4.18% and 4.04%, p = 0.023) (Figure 2).

**Table 4:** Subjects with symmetry of back shape (Mean +/- 2 SD) and severe trunk asymmetry (Beyond +/- 2 SD).

		Boys					Girls				
		**Mean +/- 2 SD**	**Beyond + 2 SD (Right)**	**Beyond – 2 SD (Left)**	**Total**	***p***	**Mean +/- 2 SD**	**Beyond + 2 SD (Right)**	**Beyond – 2 SD (Left)**	**Total**	***p***
**T (Standing)**	Count	**2705**	**57**	**64**	**2826**	0.921	**2738**	**143**	**61**	**2942**	0.072
	*Col %*	*95.72*	*2.02*	*2.26*	*100.00*		*93.07*	*4.86*	*2.07*	*100.00*	
			
**TL (Standing)**	Count	**2710**	**52**	**64**	**2826**		**2788**	**90**	**64**	**2942**	
	*Col %*	*95.90*	*1.84*	*2.26*	*100.00*		*94.77*	*3.06*	*2.18*	*100.00*	
			
**L (Standing)**	Count	**2704**	**57**	**64**	**2825**		**2830**	**73**	**39**	**2942**	
	*Col %*	*95.72*	*2.02*	*2.27*	*100.00*		*96.19*	*2.48*	*1.33*	*100.00*	

**T (Sitting)**	Count	**2659**	**112**	**53**	**2824**	0.020	**2744**	**147**	**50**	**2941**	0.009
	*Col %*	*94.16*	*3.97*	*1.88*	*100.00*		*93.30*	*5.00*	*1.70*	*100.00*	
			
**TL (Sitting)**	Count	**2671**	**84**	**70**	**2825**		**2751**	**120**	**70**	**2941**	
	*Col %*	*94.55*	*2.97*	*2.48*	*100.00*		*93.54*	*4.08*	*2.38*	*100.00*	
			
**L (Sitting)**	Count	**2625**	**110**	**89**	**2824**		**2757**	**112**	**72**	**2941**	
	*Col %*	*92.95*	*3.90*	*3.15*	*100.00*		*93.74*	*3.81*	*2.45*	*100.00*	

Comparison of ATIs beyond 2 SDs in standing and sitting FB positions for each gender by spinal region and side.

### Comparison of severe TA in pre- and post-menarcheal girls

Table 5 shows that there is little or no difference in the severity of the severe TAs between boys and girls for position and laterality.

**Table 5:** Subjects with severe trunk asymmetry.

			**Gender**				**Total**		
			**Boys**		**Girls**		**Mean**	**Std Deviation**	
			**Mean**	**Std Deviation**	**Mean**	**Std Deviation**			**p**
Standing	T	Right	7.74	1.01	8.21	1.91	8.09	1.75	0.267

		Left	7.91	1.92	9.00	1.70	8.43	1.86	0.183

	TL	Right	8.56	3.01	8.55	2.04	8.55	2.42	0.982

		Left	8.03	1.32	9.15	2.40	8.56	1.98	0.016

	L	Right	8.03	1.30	8.14	1.64	8.10	1.51	0.733

		Left	8.06	1.66	13.24	12.44	9.86	7.72	0.107

Sitting	T	Right	7.87	1.25	8.54	1.80	8.35	1.68	0.132

		Left	Not feasible						

	TL	Right	8.67	1.39	8.77	2.44	8.74	2.21	0.859

		Left	8.31	1.40	8.06	1.48	8.19	1.42	0.627

	L	Right	8.43	1.89	8.93	2.35	8.68	2.13	0.367

		Left	9.35	3.64	8.18	1.72	8.89	3.05	0.330

Comparison of mean values of TA by gender, spinal region and laterality.

### Severe TA – comparisons by relatively lower and relatively higher BMIs

**Figure 6:**
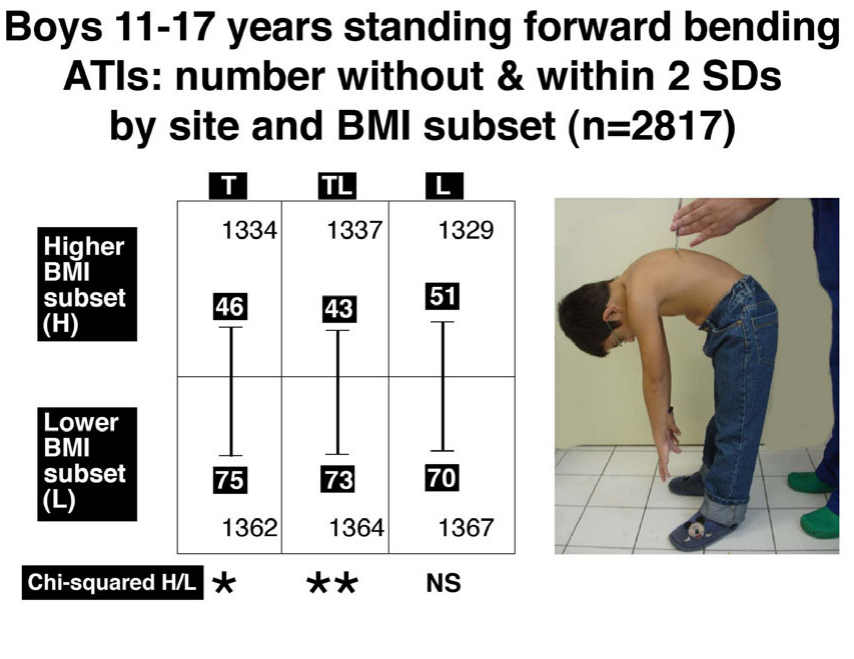
**Boys 11–17 years**. Standing forward bending position. Angle of trunk inclinations, number outside and within 2 standard deviations by site and BMI subset (n = 2817). Note there is significantly more severe TA in the lower BMI subset relative to the higher BMI subset for thoracic (T) and thoracolumbar (TL) but not lumbar (L) regions. * = 0.01 < P < 0.05, ** = .001 < P < 0.01, NS = not significant.

**Figure 7:**
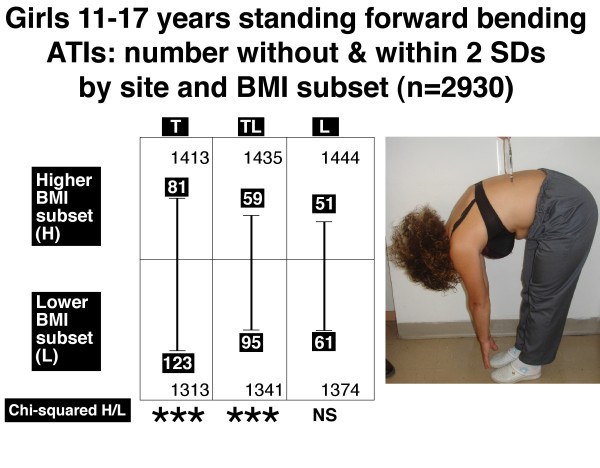
**Girls 11–17 years**. Standing forward bending position. Angle of trunk inclinations, number outside and within ± 2 standard deviations by site and BMI subset (n = 2930). Note there is significantly more severe TA in the lower BMI subset relative to the higher BMI subset for thoracic (T) and thoracolumbar (TL) but not lumbar (L) regions. *** = P < 0.001, NS = not significant.

**Figure 8:**
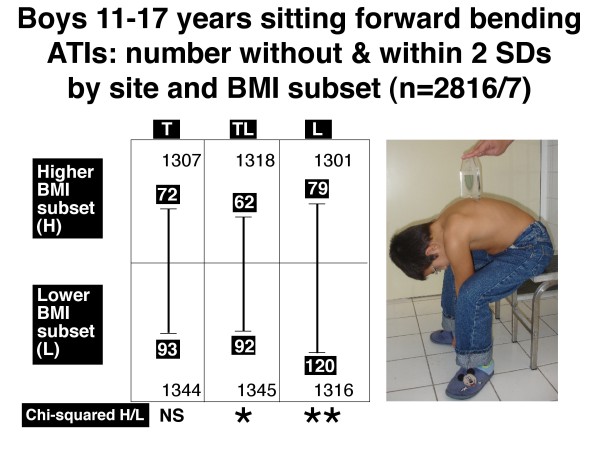
**Boys 11–17 years**. Sitting forward bending position. Angle of trunk inclinations, number outside and within 2 standard deviations by site and BMI subset (n = 2816/7). Note there is significantly more severe TA in the lower BMI subset relative to the higher BMI subset for thoracolumbar (TL) and lumbar (L) but not thoracic (T) region. * = 0.01 < P < 0.05, ** = .001 < P < 0.01, NS = not significant.

**Figure 9:**
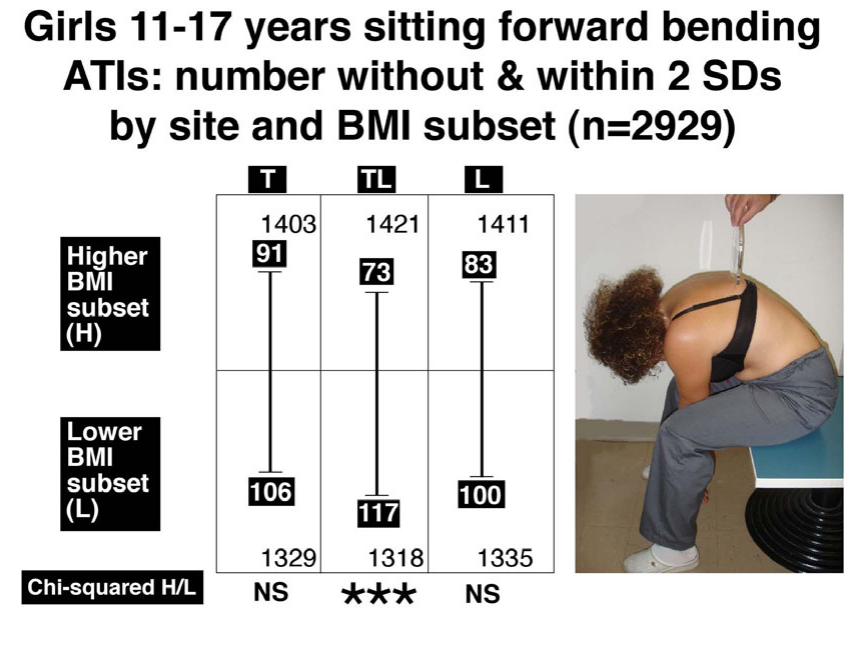
**Girls 11–17 years**. Sitting forward bending position. Angle of trunk inclinations, number outside and within 2 standard deviations by site and BMI subset (n = 2929). Note there is significantly more severe TA in the lower BMI subset relative to the higher BMI subset for thoracolumbar (TL) but not thoracic (T) and lumbar (L) regions. *** = P < 0.001, NS = not significant.

Figures 6, 7, 8 and 9 show the number of boys and girls with ATIs outside 2 SDs in each of the three regions, thoracic, thoracolumbar and lumbar, in standing and sitting FB positions separately for those with relatively higher and relatively lower BMIs.

The relatively lower BMI subsets compared with the relatively higher BMI subsets show:

• *in the standing FB position*, for each of boys (Figure 6, thoracic and thoracolumbar) and girls (Figure 7, thoracic and thoracolumbar), an excess of severe TA at each of these two levels.

• *in the sitting FB position*, an excess of severe TA at each of two levels in boys (Figure 8, thoracolumbar and lumbar) and one level in girls (Figure 9, thoracolumbar).

### BMIs and association with severe TA

Table 6 shows that in the sitting FB position after correcting for age, BMI is associated significantly and negatively with thoracic TA in boys (p = 0.001) and girls (p = 0.030), i.e smaller BMIs are associated with more *severe TAs*.

**Table 6:** All subjects

**Position**	**Dependent variable**	**Explanatory variable**	**Unstandardised beta coefficient**	**[95% Conf.**	**Interval]**	***p***	**Unstandardised beta coefficient**	**[95% Conf.**	**Interval]**	***p***
			**Boys**				**Girls**			
			
**Standing position**	**ATI T**	BMI	-0.01	-0.03	0.01	0.366	-0.02	-0.05	0.00	0.034
		Age	0.05	0.00	0.10	0.077	0.05	-0.01	0.11	0.089
		Constant	-0.21	-0.92	0.51	0.566	0.34	-0.51	1.18	0.435
		
	**ATI T-L**	BMI	0.01	-0.02	0.03	0.604	-0.02	-0.05	0.01	0.142
		Age	0.03	-0.03	0.10	0.335	0.03	-0.04	0.10	0.444
		Constant	-0.33	-1.26	0.61	0.495	0.51	-0.51	1.53	0.323
		
	**ATI L**	BMI	0.01	-0.01	0.03	0.306	-0.03	-0.06	-0.01	0.009
		Age	0.00	-0.06	0.06	0.915	0.00	-0.06	0.07	0.930
		Constant	-0.07	-0.91	0.78	0.879	1.05	0.10	2.00	0.031

**Sitting position**	**ATI T**	BMI	-0.02	-0.04	-0.01	0.001	-0.02	-0.04	0.00	0.030
		Age	0.06	0.02	0.10	0.004	0.08	0.04	0.13	<0.001
		Constant	-0.02	-0.58	0.54	0.946	-0.32	-0.97	0.32	0.326
		
	**ATI T-L**	BMI	-0.01	-0.03	0.01	0.298	0.00	-0.02	0.02	0.727
		Age	0.05	0.00	0.10	0.042	0.06	0.01	0.12	0.029
		Constant	-0.23	-0.95	0.48	0.527	-0.40	-1.20	0.40	0.331
		
	**ATI L**	BMI	-0.02	-0.04	-0.01	0.013	0.00	-0.02	0.02	0.863
		Age	0.01	-0.04	0.06	0.762	0.01	-0.04	0.06	0.775
		Constant	0.66	-0.08	1.40	0.081	0.24	-0.47	0.94	0.514

Regression of BMI on angle of trunk inclination (ATI) with adjustment for age (age constant) in standing and sitting FB positions by gender and spinal region.

In addition, a significant inverse association between BMI and humps was found in boys (lumbar region, sitting FB position, p = 0.013) and in girls (thoracic, lumbar regions in standing FB position, p = 0.034, p = 0.009, respectively).

### Menarcheal age and BMI

Overall, the mean age (and standard deviation) at menarche was 11.82 ± 1.26. Menarcheal age is significantly earlier in the higher BMI subset, than in the lower BMI subset (mean and SD respectively 11.61 ± 1.26 years and 12.10 ± 1.21 years, p < 0.001, n = 735 and 982 respectively = 1717) (overall premenarcheal 1221, 517 and 704 in higher and lower BMI subset respectively).

### Comparison of severe TA in pre- and post-menarcheal girls

Table 7 shows significantly more right than left humps in the sitting FB position in the lumbar region of postmenarcheal girls (p = 0.022), to be contrasted with an equal distribution of right and left TAs of premenarcheal girls in the lumbar region.

**Table 7:** Girls with severe trunk asymmetry

		Menarche						
		No			Yes			
		Count	%	Total	Count	%	Total	*p*
T (Standing)	Beyond +/- 2 SD (Right)	72	5.85	1230	71	4.15	1712	0.088
	Beyond +/- 2 SD (Left)	23	1.87		38	2.22		

TL (Standing)	Beyond +/- 2 SD (Right)	42	3.41	1230	48	2.80	1712	0.505
	Beyond +/- 2 SD (Left)	24	1.95		40	2.34		

L (Standing)	Beyond +/- 2 SD (Right)	38	3.09	1230	35	2.04	1712	0.077
	Beyond +/- 2 SD (Left)	12	0.98		27	1.58		

T (Sitting)	Beyond +/- 2 SD (Right)	66	5.37	1229	81	4.73	1712	0.641
	Beyond +/- 2 SD (Left)	19	1.55		31	1.81		

TL (Sitting)	Beyond +/- 2 SD (Right)	58	4.72	1230	62	3.62	1711	0.212
	Beyond +/- 2 SD (Left)	33	2.68		37	2.16		

L (Sitting)	Beyond +/- 2 SD (Right)	33	2.69	1229	79	4.61	1712	0.022
	Beyond +/- 2 SD (Left)	33	2.69		39	2.28		

Numbers premenarcheal and postmenarcheal in standing and sitting FB positions by back hump laterality and spinal region.

## Discussion

### Intrinsic back shape asymmetry (Figure 2, Table 4)

The girls and boys in the sitting FB position show a similar magnitude of severe TAs with significantly more TAs on the right (Figure 2). The relative frequency of severe TAs is significantly higher in girls than boys for each of the right thoracic and thoracolumbar regions (Figure 2). There is little or no difference in the magnitude of severe TAs between boys and girls (Table 5).

### Body mass index (BMI) in children screened for scoliosis

Our findings confirm that BMI is normal in children screened for scoliosis (Figure 3) [7,16,23-25]. BMI is reported to be normal before scoliosis develops [24,25]. While in general use as an indicator of adiposity and readily calculated, BMI needs further evaluation [57].

### Relatively earlier menarche with relatively higher BMIs

The earlier menarche of the higher relative to the lower BMI subset confirms findings for scoliosis screening referrals [23]. Several studies of normal girls show a relationship between BMI and measures of pubertal onset, and girls with relatively higher BMI are more likely to have earlier menarche [58,59]. Our findings are consistent with knowledge of a link between body fat and the timing of puberty with (1) *leptin *playing a permissive role [58,59], and (2) *kisspeptin *providing a critical metabolic signal initiating puberty through pulsatile GnRH secretion [59-62] by activating the G-protein coupled receptor-54 [61]. BMI in girls is said to be a good although imperfect surrogate measure of body fat [59]. In boys, few studies have found a link between body fat and earlier puberty [59]. We have no puberty maturity data for boys by which to asses whether puberty was earlier in their higher BMI subset.

### How may the lower BMI subset of girls with later menarche be related to the excess of severe TAs in the sitting FB position?

The girls with *relatively lower BMI *are associated statistically with each of (1) significantly later menarche, (2) excess of premenarcheal girls, and (3) significant excess of severe TA (Figures 8 &9). Smaller BMIs are associated with more *severe TAs *after correcting for age (Table 6). The linkage of lower BMI with TA has not been reported, but it extends observations on girls with AIS and *relatively lower BMI*, namely [23,30]:

(1) girls with right thoracic AIS show statistically significant associations of each of Cobb angle and apical vertebral rotation with upper limb length asymmetry (upper arms); and

(2) preoperative girls show upper arm length asymmetry significantly greater than in screened and normal girls.

These observations and other evidence from screened and normal girls suggested that girls with AIS have a dysfunctional energy balance involving the hypothalamus [23,27-30]. Bodily energy reserves are managed actively by powerful and unconscious complex systems that regulate food intake, substrate partitioning, and energy expenditure [63]. The complex systems include white adipose tissue, the adipose-tissue derived hormone leptin (and other cytokine-hormones), hypothalamus, and neuroendocrine axes including the sympathetic nervous system [63,64]. Energy balance (bioenergetics) is known to influence bone formation, resorption and bone growth in mice [64-66], and there is evidence suggesting it does so in children [23,30,67].

We apply the hypothesis of pathogenesis proposed for girls with AIS [23,30] to girls and boys with severe TAs, namely: TAs are caused by a *genetically-determined selectively increased hypothalamic sensitivity (up-regulation) to lepti*n with asymmetry as an adverse response *(LHS concept)*. This hypothalamic functional asymmetry is expressed bilaterally via the sympathetic nervous system mainly to the growing trunk to produce left-right asymmetry in ribs and/or vertebrae leading to severe TAs, when beyond the capacity of postural mechanisms of the somatic nervous system to control the shape distortion in the trunk (*neuro-osseous escalator concept*) [68].

The evidence of Qiu et al [21] confirmed by Moreau (Dr A Moreau personal communication), suggests that the *lower BMI subsets *of the girls and boys in our study had relatively *lower circulating leptin levels *than those with relatively higher BMIs. If so, the lower circulating leptin levels may have exacerbated the hypothalamic processes including asymmetry leading to the excess of severe TA in the lower BMI subsets.

We suggest that the majority of girls and boys with TA who do not progress to AIS [24], may have less severe involvement of their autonomic and somatic nervous systems. They may also lack hormonal changes [30] and osteopenia [69,70] posited to contribute to the curve severity of preoperative girls with AIS [30] in the biomechanical spinal growth modulation of progressive AIS [71-73].

### LHS concept, melatonin-signaling pathway dysfunction, osteopontin and sCD44

In progressive AIS, Moreau and colleagues [74,75] reported melatonin-signaling transduction to be impaired in osteoblasts, myoblasts and lymphocytes, caused by the dysfunction of G inhibitory (Gi) proteins. This mechanism does not appear to explain:

• the association of an excess of severe TA with *relatively lower BMI *suggesting a link with energy homeostasis (this paper); and

• disturbance in the autonomic nervous system regulating blood flow to the anterior chest wall in girls with right thoracic AIS [76-79] – unless the sympathetic nervous system also has Gi-signaling defect, or induces a Gi-signaling defect in growth plates and bone (see below).

Most recently, Moreau et al [80,81] report mean plasma *osteopontin (OPN) *levels are increased in patients with (1) idiopathic scoliosis, correlating significantly with curve severity, and in (2) "an asymptomatic at-risk group" (offspring born from at least one scoliotic parent). In contrast, mean plasma levels of soluble CD44 receptor (sCD44) are significantly lower in patients with Cobb angles of 45 degrees or more [80,81]. No *OPN *or sCD44 data are published for non-familial TA with or without spinal deformity.

### Age at which severe TA become detectable in girls and boys

TAs in normal children become evident in juvenility, earlier in girls than boys [45]. Normal juveniles are more symmetric in back shape than are adolescents with girls more symmetric than boys. Increase in TA takes place in girls at 6–7 and 8–9 years and in boys at 8–9 years [45]. We suggest this developing TA involves energy balance controlled by the hypothalamus starting its preparations for puberty.

The greater susceptibility of girls than boys to progressive AIS is attributed to a greater *down-regulation (i.e. central resistance) to leptin *of the female hypothalamus established in hominin evolution [28,30]. Hence, hypothalamic *up-regulation (i.e central sensitivity) *with its asymmetries contributing to AIS should be greater in girls than boys. Consistent with this prediction is finding that the relative frequency of severe TAs is significantly higher in girls than boys for the right thoracic and thoracolumbar regions [Figure 2 and Table 4 where right relative to left ATIs outside 2 SDs in both boys (p = 0.020) and girls (p = 0.009)], but the girls' severe TAs are not more severe than those of the boys (Table 5).

### Musculo-skeletal mechanisms in the trunk that may determine TAs in normal girls and boys

Mechanical factors involving ribs and/or vertebrae and spinal cord during growth may localize AIS to the thoracic spine [30] and contribute to its sagittal spinal shape alterations [82-84]. The musculo-skeletal mechanisms that may initiate TAs – thoracic, thoracolumbar and lumbar of normal adolescents are unknown. It is improbable that either relative anterior spinal overgrowth [82-86] or asynchronous spinal neuro-osseous growth [26,87-89] could determine TAs. Possibilities include dorsal shear forces, with axial vertebral rotation [44,52,53] and neuromuscular mechanisms acting dysfunctionally on vertebral and rib growth. More likely are growth processes involving ribs [9,90] with the rib cage deformity preceding the spinal deformity [48]. In the normal adult, pre-existing thoracic axial vertebral rotation, usually to the right, has been demonstrated and accounted for by asymmetrical anatomy of thoracic organs [52]. This axial vertebral rotation may be contributed to by asymmetrical growth in one or more neurocentral synchondroses [90-94], vertebral body torsion [95,96] and/or ribs [9,47,48,76,90,97-100].

### Thoraco-spinal pathogenetic concept for right thoracic (RT) AIS in girls

The *LHS concept *extends, and provides a wider scientific framework for, the *thoraco-spinal pathogenetic concept *for right thoracic (RT) AIS of girls [76,97,98]. A longitudinal study of anterior chest wall blood supply of girls with progressive right thoracic (RT) AIS, supports the view that RT AIS in females is associated with a disturbance in the autonomic nervous system regulating blood flow to the anterior chest wall [77,78]. The *thoraco-spinal concept *is supported by recent peripheral nerve studies [79].

### Lower spine AIS and hormesis

In lower spine AIS (thoracolumbar and lumbar curves), iliac height is greater on the curve concavity [101,102]. In AIS girls with such curves, iliac height asymmetry and apical vertebral rotation, each correlate positively with BMI like the 'dose' of a hormetic effect [103]. Given that BMI may be a surrogate measure for leptin [21], these findings are consistent with the phenomenon of *hormesis *[104]; that is a *bimodal dose response*, first stimulation and then an adverse response, usually inhibition, to drugs, toxins and a hormone such as leptin. In rats, infused leptin increases sympathoactivation in a dose-dependent manner suggesting that leptin may act hormetically on the normal rat hypothalamus [105]. In AIS girls with lower spine scoliosis, *the LHS concept *postulates that the left-right iliac length asymmetries result from an adverse *hormetic response to leptin *in the hypothalamus [103], a suggestion considered plausible [Calabrese E, personal communication].

### Rett syndrome

In the Rett syndrome, raised circulating leptin levels and overactivity of the sympathetic nervous system are pathophysiological features of this genetic neurodevelopmental disorder [106]. In patients with Rett syndrome, the skin sympathetic responses were reported to show relatively lower amplitude on the foot ipsilateral to the convex side of the scoliosis [107]. Skin sympathetic responses need studying in AIS girls with the recording electrodes placed on both sides of the trunk and at other sites [30].

### Test of the hypothesis

A test of the autonomic component of the hypothesis testing skin sympathetic responses [107] in students with severe TAs is suggested.

## Conclusion

(1) The relation between truncal asymmetry (TA), body mass index (BMI) and menarcheal status is evaluated in healthy adolescents age 11–17 years (boys 2817, girls 2930) (Table 1).

(2) Each child was assigned a value of *"lower" or "higher" BMI *relative to median values constructed for each age group, gender, position and spinal region.

(3) The sitting FB position is thought to express intrinsic TA free from extrinsically-induced effects of any leg-length inequality. The analyses of the findings for the pathogenetic interpretation utilise these data.

(4) In the sitting FB position for severe TA:

a) There is no sex difference in TAs for any of the three spinal regions.

b) An excess of right relative to left ATIs outside 2 SDs in both boys (p = 0.020) and girls (p = 0.009).

c) The relative frequency of severe TAs is significantly higher in girls than boys for each of the right thoracic and thoracolumbar regions (Figure 2 and Table 4).

d) After correcting for age, smaller BMIs associated with more *severe TAs *in boys and girls (Table 6).

(5) Mean age at menarche was significantly later in the lower BMI subset, than in the higher BMI subset (mean and SD respectively 12.10 ± 1.21 years, and 11.61 ± 1.26 years, p < 0.001).

(6) The girls with relatively lower BMI are associated with significantly later menarche, and a significant excess of TAs. These observations together with other evidence [23,30], suggests a relation of trunk asymmetry to energy balance through the hypothalamus.

(7) As with a recent hypothesis for the pathogenesis of AIS in girls [27,30], we suggest that severe TAs are caused by a *genetically-determined selectively increased sensitivity (up-regulation, i.e. increased sensitivity) of the hypothalamus to lepti*n with asymmetry as an adverse response to stress, increased by lower circulating leptin levels associated with relatively lower BMIs *(LHS concept)*. This hypothalamic functional asymmetry is expressed via the sympathetic nervous system bilaterally to produce left-right asymmetry in ribs and/or vertebrae leading to severe TA, when beyond the capacity of postural mechanisms of the somatic nervous system to control the shape distortion in the trunk.

(8) We suggest that the majority of girls and boys with TA who do not progress to AIS [24], may have less severe involvement of their autonomic and somatic nervous systems. They may also lack hormonal changes [30] and osteopenia [69] both of which may contribute to the curve severity of preoperative girls with AIS [30] in the biomechanical spinal growth modulation of progressive AIS [71-73].

## Competing interests

The authors declare that they have no competing interests.

## Authors' contributions

TBG proposed the idea of the presented study, performed part of the literature review, contributed to the interpretation of data and in drafting the manuscript. RGB guided parts of the analysis, contributed to the literature review, interpreting the data and drafting the manuscript. CM contributed in the statistical analysis and drafting the manuscript. ESV performed part of the literature review and participated in the school screening program. GT and AK participated in the school screening program. All authors read and approved the final manuscript.
